# Development and validation of a mobile application for heart failure patients self-care

**DOI:** 10.1590/1980-220X-REEUSP-2022-0315en

**Published:** 2023-01-06

**Authors:** Mailson Marques de Sousa, Camila Takáo Lopes, André Atanasio Maranhão Almeida, Taciana da Costa Farias Almeida, Bernadete de Lourdes André Gouveia, Simone Helena dos Santos Oliveira

**Affiliations:** 1 Universidade Federal da Paraíba, Departamento de Enfermagem Clínica, João Pessoa, PB, Brazil; 2 Universidade Federal de São Paulo, Escola Paulista de Enfermagem, São Paulo, SP, Brazil; 3 Instituto Federal da Paraíba, Curso Técnico de Informática, Esperança, PB, Brazil; 4 Universidade Federal de Campina Grande, Unidade Acadêmica de Enfermagem, Campina Grande, PB, Brazil; 5 Universidade Federal de Campina Grande, Unidade Acadêmica de Enfermagem, Cuité, PB, Brazil; 6 Universidade Federal da Paraíba, Escola Técnica de Saúde, João Pessoa, PB, Brazil

**Keywords:** Heart Failure, Self Care, Mobile Applications, Educational Technology, Validation Study, Insuficiencia Cardíaca, Autocuidado, Aplicaciones Móviles, Tecnología Educacional, Estudio de Validación, Insuficiência Cardíaca, Autocuidado, Aplicativos Móveis, Tecnologia Educacional, Estudos de Validação

## Abstract

**Objective:**

To develop and verify content and face validity evidence of a mobile application prototype for heart failure patients self-care.

**Method:**

Study of technological development based on Contextualized Instructional Design. In the validation stage, six cardiology specialist nurses evaluated the content through the *Suitability Assessment of Materials* and 13 people with heart failure underwent face validity assessment, through content validity index. Data were analyzed using descriptive statistics.

**Results:**

The prototype of the application, called “Tum Tum”, has an interface and free navigation screens covering the concept of heart failure, its causes, symptoms and signs, diagnosis, and treatment. It has a mosaic with educational guidelines, resources for early recognition of signs of clinical decompensation, registration for body weight control, reminders for medication use, consultation and exam schedule. The technology proved to be adequate among specialists and the target audience.

**Conclusion:**

It was possible to develop and validate the content and face of the “Tum Tum” application prototype, which demonstrates the potential to promote self-care in people with heart failure.

## RESUMO

**Objetivo:**

Desenvolver e verificar as evidências de validade de conteúdo e face de um protótipo de aplicativo móvel para o autocuidado de pessoas com insuficiência cardíaca.

**Método:**

Estudo de desenvolvimento tecnológico baseado no Design Instrucional Contextualizado. Na etapa de validação, seis enfermeiros especialistas em cardiologia avaliaram o conteúdo por meio do *Suitability Assessment of Materials* e 13 pessoas com insuficiência cardíaca realizaram avaliação de face, pelo índice de validade de conteúdo. Os dados foram analisados por meio de estatística descritiva.

**Resultados:**

O protótipo do aplicativo, nomeado “Tum Tum”, possui interface e telas de livre navegação que abordam o conceito da insuficiência cardíaca, causas, sinais e sintomas, diagnóstico e tratamento. Apresenta mosaico com orientações educativas, recursos para o reconhecimento precoce de sinais de descompensação clínica, registro para controle do peso corporal, lembretes para o uso de medicamentos, agenda de consultas e exames. A tecnologia mostrou-se adequada entre especialistas e público-alvo.

**Conclusão:**

Foi possível desenvolver e validar o conteúdo e face do protótipo do aplicativo “Tum Tum”, que demonstra potencial para promover o autocuidado em pessoas com insuficiência cardíaca.

## RESUMEN

**Objetivo:**

Desarrollar y verificar evidencias de validez de contenido y facial de un prototipo de aplicación móvil para el autocuidado de personas con insuficiencia cardíaca.

**Método:**

Estudio del desarrollo tecnológico basado en el Diseño Instruccional Contextualizado. En la etapa de validación, seis enfermeros especialistas en cardiología evaluaron el contenido a través del *Suitability Assessment of Materials* y 13 personas con insuficiencia cardíaca se sometieron a evaluación facial, por el índice de validez de contenido. Los datos fueron analizados utilizando estadística descriptiva.

**Resultados:**

El prototipo de la aplicación, denominado “Tum Tum”, cuenta con una interfaz y pantallas de navegación gratuitas que abordan el concepto de insuficiencia cardiaca, causas, signos y síntomas, diagnóstico y tratamiento. Cuenta con un mosaico con pautas educativas, recursos para el reconocimiento temprano de signos de descompensación clínica, registro para el control de peso corporal, recordatorios para el uso de medicamentos, calendario de consultas y exámenes. La tecnología demostró ser adecuada entre los especialistas y el público objetivo.

**Conclusión:**

Se logró desarrollar y validar el contenido y la face del prototipo de la aplicación “Tum Tum”, que demuestra el potencial para promover el autocuidado en personas con insuficiencia cardíaca.

## INTRODUCTION

Heart failure (HF) is a chronic disease with a high prevalence worldwide, estimated at 64.34 million people^(1)^, responsible for low quality of life and morbidity and mortality, high rates of hospitalizations due to decompensation and, consequently, high financial costs for the health system^(2)^.

Considering the complexity of the treatment, it is imperative that patients with HF adopt self-care behaviors to maintain compensation. Better levels of self-care are related to lower readmission rates^(3)^, better quality of life^(4)^ and lower mortality^(5)^. However, poor adherence to self-care is a worldwide problem that predicts unfavorable clinical outcomes^(6,7)^. In Brazil, the scarcity of specialized clinics in health care settings and gaps in patients’ knowledge about the disease and treatment contribute to the low adherence of patients with HF to the complex therapeutic regimen^(8)^.

The use of technological resources can be a viable strategy to strengthen healthy behaviors and promote better adherence to treatment. Mobile health technology (*mHealth*) is a promising tool that can help improve self-management of chronic diseases. It is defined as the use of smartphones, tablets, and other mobile devices to provide health care and preventive health services^(9)^.

The mobile applications (apps) have been used as an adjuvant educational tool for people with HF, to improve the information and education systems in different aspects, promote a positive perception regarding the importance of self-care, helping the patient to self-monitor symptoms and signs, control and manage the disease, with no time and space restrictions^(10,11)^. International evidence points to beneficial results regarding the use of *mHealth* technology as an intervention strategy for self-care and reduction in hospitalization rates^(12,13)^.

Despite the popularity of smartphones and the use of mobile devices in the world, the strategy *mHealth* in Brazil is incipient, given that the clinical care of people with HF is heterogeneous in view of economic, educational, and cultural differences, as well as in access to health services.

When searching for apps in the Google® Play Store and Apple® Store, no apps aimed at promoting self-care in people with HF in Portuguese were found. The apps available are from North American countries in the English language. The use of smartphones by the population with HF is increasingly frequent^(14)^, which highlights the pertinence of building complementary tools via mobile applications and their potential usefulness for health self-management.

Thus, it becomes relevant to develop innovative technologies for the promotion of self-care, which can be easily incorporated into clinical practice to promote knowledge, self-management skills, and to improve health-related quality of life to reduce hospitalizations, emergency readmissions, and mortality in this population. This proposal is in line with the Sustainable Development Goals (SDGs) in terms of health and well-being. In view of the above, the objective is to develop and verify the evidence of content and face validity of a mobile application prototype to promote self-care in people with HF.

## METHOD

### Design of Study

The methodology of this study is technological, based on the Contextualized Instructional Design (CID). This method involves the following steps: analysis; design/development; implementation; and evaluation^(15)^.

### Local

The study was carried out in a large federal teaching hospital located in the state of Paraíba, a reference in specialized cardiology care.

### Study Protocol

The study was carried out in three steps, namely:

#### 1st Step – Analysis

This step consisted of providing tools and resources to meet the learning needs of the target audience^(15)^. For that, a narrative review of the literature was carried out to identify the educational content directed to the self-care of people with HF. Pubmed/MEDLINE, CINAHL, and Scopus databases were consulted in search of scientific productions. The following descriptors of *Medical Subject Headings* (MeSH) were used: “Heart failure”; “Self-care”; and “Mobile applications”, associated through the Boolean operator “AND”.

Based on scientific evidence and researchers’ experience in cardiovascular care, remote scientific meetings were held to prepare the textual content and select educational topics on non-pharmacological and pharmacological management indicated for people with HF, such as: daily monitoring of body weight, restriction of fluids, consumption of a low-sodium diet, correct and regular use of prescribed medications, practice of physical activity, recognition of signs of clinical decompensation, immunizations, among others^(3,16)^.

#### 2nd Step – Design/Development

In this stage, the instructional content was elaborated to promote self-care, using the literature evidence identified in the first stage. Accessible language was adopted, without the use of technical terms, to help in understanding the content. There was help from a consultant, a graphic designer, for creating and developing the interface, and from a systems analyst, for implementing the prototype. Periodic meetings with the project team were held to discuss name, interface, screens, icons, layout, color selection, illustrations, typography, font size, spacing, colors, image, figures, and prototype functionality positioning.

The interface and screens were created using the software Figma® that, through the wireframe, simulated the prototype of the application. The icons and illustrations were customized by the design professional and/or adapted from two Open Source libraries: Storyset and Feather Icons, in shades of navy blue and red, in reference to blood circulation, on a white background and Milush typography. Easy-to-understand illustrations were used for better comprehension of the text.

Currently, in the smartphones market, there are essentially two platforms that dominate the market, Android® and iOS® (used in *iPhones*). At first, it would be necessary to implement an application to meet each of them, if desired, and each one would require specific software, languages, and knowledge. However, the development of a hybrid model with a single code base was selected. Thus, in the involvement phase, more specifically in the implementation of the application prototype, the framework *React Native* was used, which allows the construction of native mobile applications for the Android® and iOS® platforms based on a single project written in JavaScript language and does not compromise the user experience. With this single base, it will be possible to make the app available in the Play Store® and Apple Store® application stores, achieving greater public access. The user will need to have access to the internet to download the application and, after downloading it on the smartphone, the application can be used offline.

This phase involved building the application prototype with the collaboration of a systems analyst, who developed the software based on the content produced in the previously described steps.

#### 3rd Step – Validation of Content and Face

Once the prototype with interface with all the content was finished, the content was evaluated and validated by an experts committee. Although the number of specialists is not consensual, the inclusion of five to 10 in the validation process is suggested^(17)^.

The identification of the expert committee occurred through the Lattes Platform. In the tab for advanced search by subject, the following keywords were used: self-care, heart failure, and health technologies. Experts were recruited through non-probabilistic and intentional sampling. The following inclusion criteria were adopted: having a graduate certificate and/or a Master’s degree in cardiovascular nursing; having clinical-assistance experience with the target audience for at least two years; and having work published in journals and/or events on the subject. Twenty-eight potential experts were identified.

The experts were invited by email. Those who agreed to participate received a questionnaire for professional characterization, an evaluation form about the technical-scientific content of the application, and the Free Informed Consent Form (FICF) through a form available via google forms with screens in PDF format and a link for access to the high fidelity prototype in the Figma® software. The material had to be returned within 20 days, with this period being extended for another 15 to 30 days according to the request of the specialists.

Next, face validation took place with the target audience, at the cardiology outpatient clinic of the teaching hospital. A non-probabilistic sample was selected for convenience, consisting of patients with a medical diagnosis of HF, of any etiology, aged 18 years or older, who had a smartphone and finished high school. The decision of adopting the criterion of finished high school was taken so that the participants could access and evaluate the interface and clarity of the content independently.

Patients with no cognitive conditions to answer the data collection instruments did not integrate the sample. To identify the preserved cognitive condition, five questions adapted from a previous study were applied^(18)^: What is today’s date? How old are you? What day of the week are we on? What is the name of the place we are in right now? What’s your full name? Patients who made mistakes or did not know how to answer three or more questions did not participate in the study. The researchers went to the cardiology outpatient clinic, having a list with the names of the people who would be assisted on that day. Those who met the inclusion criteria were invited to participate in the research, before or after a medical consultation. An Apple iPhone XR® was used with *software* Figma® for prototype visualization.

### Data Collection

Data were collected from October 2021 to February 2022. The specialists evaluated the application interface and content individually, using the adapted instrument *Suitability Assessment of Materials* (SAM) validated for Brazilian Portuguese, which assesses the difficulty and convenience of health-related educational materials^(19)^. SAM is an instrument with 22 items, organized into six categories: content; language; graphic illustrations; layout and presentation; motivation; and cultural appropriateness. As response options for each aspect evaluated, the following are scored: 2 points = Adequate, 1 point = Partially Adequate, 0 = Inadequate. In this investigation, the item “The illustrations have legends” was not considered by the specialists, since the illustrations used are self-explanatory.

The evaluation instrument intended for face validation by the target audience was adapted from a previous study carried out with people with HF^(20)^, consisting of two parts: the first, containing information for characterizing the sample, and the second, evaluative items about the application organization, illustrations, figures, font size, content, language, and motivation to use the application. The items were answered using a four-point Likert-type scale, as follows: 1 = strongly disagree; 2 = disagree; 3 = agree; 4 = strongly agree. At the end, participants were asked the following questions: Do you need any further information about self-care? What did you like about the app? What didn’t you like about the app? Do you believe you can use this application in your daily life?

### Data Analysis and Treatment

The answers obtained from the specialists and participants were organized in tables, followed by descriptive analysis, with calculation of absolute and relative frequencies, as well as measures of central tendency (mean) and dispersion (standard deviation).

The total SAM score ranges from 0 to 44 points. In this study, the possible sum is equivalent to 42 points, since the item “The illustrations have legend” was not applied. Thus, the result obtained by the experts was calculated by means of the total sum of points obtained divided by the maximum possible score and multiplied by 100. To evaluate the global SAM score, the arithmetic mean of the sums of each specialist (Σ) was calculated, divided by the number of specialists and multiplied by 100. Thus, it was possible to find the following percentages: 70 – 100% (superior material), 40 – 69% (adequate material) or 0 – 39% (inadequate material)^(21)^.

To analyze the results of the face validation by the target audience, the Content Validity Index (CVI) was used through the agreement of the items individually (Item-Level Content Validity Index I-CVI), expressed by the formula: number of participants who rated the items as 3 or 4, divided by the total number of participants. To classify the item as valid, a value ≥ 0.78 is desirable. The evaluated items that obtained CVI < 0.78 were reformulated according to the suggestions of people with HF^(22)^. To evaluate the content in general, the sum of the items with CVI > 0.78 was performed, and divided by the total number of items. Content is considered valid with a global score ≥ 0.80^(22)^. In addition, the general opinion of the participants regarding the application was requested.

### Ethical Aspects

The study complied with the ethical aspects contained in Resolution 466/12 of the National Health Council and was approved by the Research Ethics Committee, under Opinion No. 4.620.748/2021. The participants authorized their participation by signing the Free and Informed Consent Form.

## RESULTS

“Tum Tum” is an application prototype developed to provide educational information about self-care in HF. The application received this name in allusion to the sound of heartbeats. The icon representing the application is a heart.

On the home screen, the registration can be done by filling in personal information. The user can also access the content in guest mode through free navigation screens. However, to register and view data history, prior log in is required. Next, welcome screens are displayed to the user with information about the purpose and content of the app.

Once the user has logged in or opted for the guest mode, “Tum Tum” presents its interface with the functionalities. This interface has three tabs and a bottom bar, which indicates the tab selected and allows switching to another one. The first tab is called Mosaic and has five buttons at the top, which open screens with information about HF, causes, symptoms, diagnosis, and treatment. Below, an informative mosaic is displayed, organized into nine educational topics to promote self-care: (1) weight; (2) food; (3) exercises; (4) sexual activity; (5) medicines; (6) mental health; (7) sleep;(8) vaccination; and (9) smoking habit.

The second tab, called My Health, is divided into Routine and History. In the Routine, the patient finds functionalities for registering medications, exams and medical appointments. It is possible to program reminders for these according to medical prescription or as needed, and it also features functionality to inform daily body weight as well as report symptoms. The patient will be able to select signs and/or symptoms of HF through checkboxes and will be alerted to seek an urgent and emergency service when checking three or more clinical manifestations, a condition that signals a state of clinical decompensation of the disease. It is also possible to indicate the number of pillows used to sleep, a resource that allows assessment of breathing difficulties during sleep (orthopnea). In History, the user will be able to briefly observe information that he/she recorded previously, with emphasis on the graph with weight variation, which warns of sudden weight gain.

**Figure 1:**
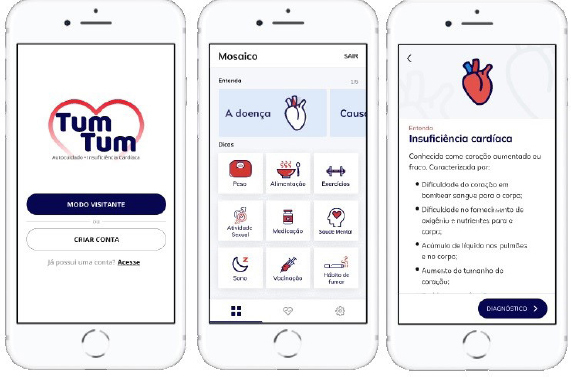
Screenshots of the “Tum Tum” graphical interface. João Pessoa, PB, Brazil, 2022

The third tab contains the Settings, which allow making adjustments more related to the needs of each user in the application and accessing information about the project. It should be noted that none of the data is mandatory, ensuring the user accessibility to all the content produced. Figure 1 illustrates the application interface.

The initial version of the prototype was sent to a committee of experts for content evaluation. Of the 28 potential experts invited, 20 did not respond to the initial contact, two declined to participate, and six experts agreed to participate in the research. The six specialists were nurses, lived in the cities of São Paulo, Rio de Janeiro, Uberlândia, and Porto Alegre, had a mean age of 38.67 (± 6.62) years, were mostly female (66.7%), were PhDs in Nursing, worked as Professors, had clinical experience in the care of people with HF, and participated in research projects in the area of care technologies, which show qualification to evaluate the technological artifact. Table 1 presents the score distribution of the application evaluation by specialist nurses based on SAM items.

**Table 1:** Adequacy of the application according to the evaluation by specialist nurses – João Pessoa, PB, Brazil, 2022.

Evaluation items	Adequate	Partially adequate	Inadequate
1. Contents			
The objective is clear, which facilitates the comprehension of the material	05	01	–
The content addresses information that helps promote self-care in heart failure	03	02	01
Content is aligned with the objective	05	01	–
Content highlights the main points of self-care in heart failure	03	03	–
2. Language			
The reading level is adequate for the reader’s comprehension	04	02	–
The writing style makes the text easier to understand	04	02	–
Information is conveyed within a clear context	04	02	–
Vocabulary uses common words	05	01	–
Learning is facilitated by topics	06	–	–
3. Graphic illustrations			
The purpose of the illustration referring to the text is clear	06	–	–
The figures/illustrations are relevant	06	–	–
Figures/illustrations are self-explanatory	06	–	–
Illustrations are self-explanatory	06	–	–
The illustrations have legends	–	–	–
4. Layout and presentation			
The organization is adequate	06	–	–
Font size and type promote pleasant reading	04	02	–
Legends are used	06	–	–
5. Motivation			
Uses the interaction	01	05	–
The guidelines are specific and give examples	04	02	–
There is encouragement for self-care	02	04	–
6. Cultural adequacy			
Material is culturally appropriate to logic, language, audience experience	05	01	–
Presents culturally appropriate images	06	–	–

Regarding the SAM score, it was found that it reached high scores, classifying educational technology as superior material (≥ 70%). The entire committee of specialists also evaluated the application as of superior category, reaching an overall percentage of 88.4%. Among the 22 items evaluated, only one expert rated the content for promoting self-care in HF as inappropriate, explaining that it was possible to add more educational information. Due to the good evaluation rates achieved, there was only one round of evaluation with specialists.

**Chart 1:**
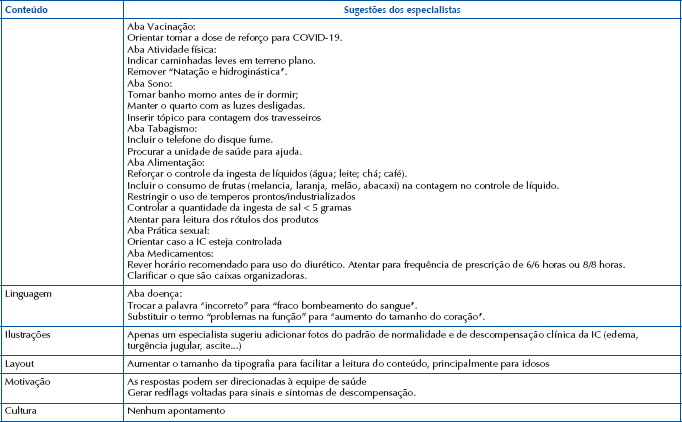
Experts’ comments and suggestions – João Pessoa, PB, Brazil, 2022.

In addition to the score established for each SAM item, the evaluators were able to use the space intended for suggestions of improvements to the prototype. The comments provided regarding the content, language, illustrations, layout, presentation and motivation for the app content are shown in Chart 1.

After adapting the material to the suggestions, face validation was carried out by the target audience. Fifteen patients with HF were invited, of which 13 agreed to participate. It should be noted that no participants had an impaired cognitive condition.

With regard to age, the mean was 55.92 (± 12.95) years, 53.8% (n = 7) were female, 38.5% (n = 5) were single, 38.5% (n = 5) were married/in a common law marriage relationship, 100% had completed high school and had an average family income of two minimum wages. The most prevalent etiology of HF was non-ischemic, with 76.9% (n = 10); 46.2% (n = 6) were in functional class III; and 30.8% (n = 4) in functional class II. The mean time since diagnosis was seven years (± 6.25), ranging from one to 21 years, and 100% were using diuretics. Asked whether they used or had used any application for HF, 92.3% (n = 12) said they had never used it. Table 2 presents the results of face validation by the target audience.

**Table 2:** Results of the face evaluation by the target audience (n = 13) – João Pessoa, PB, Brazil, 2022.

Items evaluated	CVI
1. Is the application organized?	1
2. Is the language easy to understand?	0.92
3. Are the colors and shapes of the illustrations appropriate?	1
4. Is the arrangement of figures consistent with the text?	1
5. Are the illustrations relevant to understanding the content?	1
6. Is the font size adequate?	0.69
7. Is the content enough?	0.92
8. Were you able to understand the information contained in the application?	1
9. Did you feel motivated to use this app until the end?	1
10. Do you think using this app will improve your self-care?	1

CVI = Content Validity Index.

Regarding the evaluated items, it is observed that only item 6, which evaluates the size of the letter, received a score < 0.78. The other items showed excellent agreement among the target audience, showing a total CVI of 0.98. At the end of the evaluation, participants reported: *I believe that the use of the application will help the treatment* (P1). *I will use it because I need to take care of myself* (P4). *I understand the disease better* (P7). *I liked it a lot, it has information I didn’t know* (P6). *It is important as it helps with medication* (P11). *We never know everything, we always need to know more about the heart* (P12). *I will definitely use it, it will be like a companion* (P3). No participant reported not liking the application.

## DISCUSSION

The technology *mHealth* is innovating communication processes, as it can reach larger populations, with no time restrictions, enhancing the teaching and learning process and, consequently, the promotion, prevention, therapeutic adherence, and health rehabilitation of health in different parts of the world. Studies^(23,24)^ have been carried out in high-income countries to develop a tool to support self-management of care and health education in patients with chronic diseases. In Brazil, the development of these technologies has increased; however, the high financial investment impacts its operationalization and incorporation into the public health system.

This study developed and verified content and face validity evidence of a mobile application prototype for HF patients self-care. The project was carried out in partnership with professionals from design and information technology, which ensured superior quality to the proposed technology. “Tum Tum” application interface and screens were designed with a pleasant layout, ease of use, on a white background, with interactions, clear information, accessible vocabulary, aimed at facilitating understanding and handling by people with HF. Primacy of these characteristics in the construction of “Tum Tum” considered the pertinence of the argument that mobile technologies should be built based on the elements of simplicity, usefulness, easy visualization, suitability for the target audience^(25)^.

It should be highlighted that, in the development of educational technologies, validation of content by experts and target audience is a recommended technique. Recent studies using this method obtained a similar number of participating experts^(26,27)^.

The uniqueness of nursing care in the face of human responses allowed specialists from different regions of the country to add relevant information to improve the application prototype based on the clinical experience of cardiology care. The content that should be addressed in nursing interventions was expanded towards a more congruent lifestyle for the person with HF, to favor self-care and, thus, improve health and quality of life, conferring relevance and suitability to the product designed for the public for whom it is intended. Among the recommendations, we highlight the booster for immunization against COVID-19 and the inclusion of a function that allows the sending of information to the health team, which, in its turn, allows for two-way communication.

Additionally, an expert suggested the inclusion of real illustrations with patterns of normality and clinical decompensation of HF (lower limb edema, jugular swelling and ascitic abdomen), a suggestion that was not accepted as it differs from the visual aesthetics of the illustrations adopted in the graphical interface and that would not portray educational content, but could have a negative impact on users, such as feelings of fear and anxiety.

It is important to emphasize that elements to stimulate interaction make recommendations clearer and more precise, influence the user to develop cognitive skills, and, therefore, should be considered in the elaboration of educational materials on health^(28)^. In this perspective, red flags to alert for signs of clinical decompensation, charts for monitoring body weight, alarms for medication use and consultation schedule were included. Future versions may incorporate infographics, links, animations and videos.

In this investigation, we chose to use the SAM instrument, internationally used to assess content, organization, clarity of language, use of illustrations, motivation and cultural adequacy of educational materials, to obtain educational technology consistent with the self-care needs of people with HF and with the possibility of influencing the adoption of desirable behaviors and attitudes to control the disease.

Based on the criteria established by the SAM, the application was considered superior quality technology according to the evaluation of specialist nurses. The overall score reached 88.4%, showing that the product offered is suitable for the target audience. This result is consistent with a study that built and validated a mobile application for guidance on venous thromboembolism^(28)^.

The educational guidelines addressed in the app content to encourage self-care corroborate recommendations from Brazilian^(2)^ and European^(16)^ guidelines and technologies *mHealth* ^(11,29)^ developed for patients with HF. It is believed that further studies can be conducted to update and improve this care technology, to potentiate the teaching-learning process, given the continuous advancement of scientific evidence on HF.

Studies^(20,23,26,27)^ which developed technological resources for people with chronic diseases used the CVI as a method to assess agreement in relation to the evaluated items. In this study, CVI was applied for face validation by the target audience, which allowed evaluating aesthetic aspects of the organization, interface, content, language, with evidence of adequacy being obtained, which portray the quality of the educational technology produced.

It should be noted that the typography size reached CVI < 0.78, which resulted in adjustments to meet the expectations of the lay public and facilitate reading, especially for elderly users. It is important to highlight that the participants evaluated the application prototype with the potential to help improve the comprehension of HF and the self-care actions instituted in the therapeutic regimen.

Qualitative research exploring the needs and perspectives of patients with HF for the use of health mobile technology highlighted the need to implement designs that facilitated the use based on reliable information about treatment, medications, when and how to seek emergency services, and personalized health-related reminders to strengthen self-care behaviors^(30)^. These functionalities are present in the application mentioned and may be refined as tests of communication between users and health professionals are carried out.

For professional nursing practice, the “Tum Tum” application is an innovative technology in the care of people with HF. So far, to our knowledge, this is the first nurse-led application developed in the Northeast region of Brazil. Therefore, its use shall be encouraged by nurses due to the convenience of access to expand information, add knowledge, and reinforce verbal information and, based on their records, assist health professionals in making an adequate clinical decision in the preparation of the therapeutic plan.

This study has some limitations. Although the sample of specialists complied with the literature recommendations, future research may increase the participation of other professionals from the multidisciplinary health team. Patients from a single service and with an average education level higher than the reality of users monitored in the Brazilian Public Health System are also mentioned. It is assumed that the higher the level of education, the greater the ease of use of mobile technologies. Therefore, the findings cannot be generalized and future investigations are required to evaluate the application content and functionality by people with other levels of education, aiming at reducing digital literacy barriers.

Moreover, the high financial cost is a limiting factor for the development of technologies *mHealth*. It becomes necessary to bring Nursing closer to other areas of knowledge to share information, seek low-cost solutions and care technologies that can be incorporated into clinical practice. New research will be carried out to accomplish the steps of implementation and usability evaluation not covered in this study. In addition, evaluations to verify efficiency, satisfaction, advances in the interface and possible malfunctions will be tested by users in future stages. It is expected that the technology will be soon transferred to the public sector soon, after its registration and launch on the main mobile application platforms.

## CONCLUSION

This study developed a project of graphical interface and obtained adequate evidence of content validity of the “Tum Tum” application prototype for HF patients self-care. The prototype application provides information about the disease, causes, signs and symptoms, diagnosis and treatment. Educational guidelines with essential non-pharmacological and pharmacological measures to maintain HF stability are presented. Furthermore, features for recording information, data history and red flags with signs of clinical decompensation help patients’ self-management. This innovative technological resource can be used by the multidisciplinary team as a care technology to enhance educational activities as a resource for health self-management. Future studies will be conducted to evaluate the usability and effectiveness of the technology in clinical practice.
